# Safety and Effectiveness of Chemotherapy in Elderly Biliary Tract Cancer Patients

**DOI:** 10.3390/curroncol30080524

**Published:** 2023-07-27

**Authors:** Takeshi Okamoto, Tsuyoshi Takeda, Takashi Sasaki, Tsuyoshi Hamada, Takafumi Mie, Takahiro Ishitsuka, Manabu Yamada, Hiroki Nakagawa, Tatsuki Hirai, Takaaki Furukawa, Akiyoshi Kasuga, Masato Ozaka, Naoki Sasahira

**Affiliations:** 1 Department of Hepato-Biliary-Pancreatic Medicine, Cancer Institute Hospital of Japanese Foundation for Cancer Research, 3-8-31 Ariake, Koto-ku, Tokyo 135-8550, Japan; tsuyoshi.takeda@jfcr.or.jp (T.T.); takashi.sasaki@jfcr.or.jp (T.S.); tys.hamada@gmail.com (T.H.); takafumi.mie@jfcr.or.jp (T.M.); takahiro.ishitsuka@jfcr.or.jp (T.I.); manabu.yamada@jfcr.or.jp (M.Y.); hiroki.nakagawa@jfcr.or.jp (H.N.); tatsuki.hirai@jfcr.or.jp (T.H.); takaaki.furukawa@jfcr.or.jp (T.F.); akiyoshi.kasuga@jfcr.or.jp (A.K.); masato.ozaka@jfcr.or.jp (M.O.); naoki.sasahira@jfcr.or.jp (N.S.); 2 Department of Gastroenterology, Graduate School of Medicine, The University of Tokyo, 7-3-1 Hongo, Bunkyo-ku, Tokyo 113-8654, Japan

**Keywords:** age, cholangiocarcinoma, monotherapy, combination chemotherapy, tolerability, gemcitabine, cisplatin, S-1

## Abstract

The safety and effectiveness of chemotherapy in elderly patients with biliary tract cancer (BTC) remain unclear. Therefore, we retrospectively reviewed patients who underwent chemotherapy for locally advanced, metastatic, or recurrent BTC at our institution from January 2016 to December 2021. Of the 283 included patients, 91 (32.5%) were aged 75 years or older when initiating chemotherapy. Elderly patients were more likely than non-elderly patients to receive monotherapy with gemcitabine or S-1 (58.7% vs. 9.4%, *p* < 0.001) and were less likely to experience grade 3–4 toxicities (55.4% vs. 70.2%, *p* = 0.015). The rates of termination due to intolerance (6.5% vs. 5.8%, *p* = 0.800) and transition to second-line chemotherapy (39.1% vs. 40.3%, *p* = 0.849) were similar between groups. In the overall cohort, age was not an independent predictor of overall survival (OS). Within the elderly cohort, there were no differences in severe adverse events between patients receiving monotherapy and combination therapy (50.0% vs. 63.2%, *p* = 0.211). Median OS was longer in the combination therapy group (10.4 vs. 14.1 months; *p* = 0.010); however, choice of monotherapy was not an independent predictor of overall survival. Monotherapy appears to be a viable alternative in selected elderly BTC patients.

## 1. Introduction

Biliary tract cancer (BTC) is a collective term that refers to a heterogenous group of malignancies arising in the biliary tree, including intrahepatic and extrahepatic (perihilar or distal) cholangiocarcinomas, gallbladder cancer, and sometimes ampullary cancer [1,2]. BTC is primarily a disease of the elderly. As of 2019 in Japan, 89% of patients diagnosed with BTC were aged 65 years or older, 77% were 70 years or older, 64% were 75 years or older, 47% were 80 years or older, and 28% were 85 years or older [3]. There is a similar trend worldwide; for example, the incidence of gallbladder cancer peaks at 85–89 years of age in the United Kingdom [4,5].

While only surgical resection offers a chance for cure, a large majority of BTC cases are unresectable at diagnosis [6]. Chemotherapy must therefore be considered, even in elderly cases. With one notable exception [7], most clinical trials for BTC have avoided imposing an upper age limit to its participants, focusing instead of Eastern Cooperative Oncology Group (ECOG) performance status (PS) [8,9,10,11,12,13,14,15,16,17]. Nevertheless, the median age of included patients is generally about 65 years old, with very little information for patients older than the age of 75 years. We therefore conduct this study to (1) investigate the outcomes of chemotherapy for elderly (aged 75 years and over) and non-elderly BTC patients in the real-world setting and (2) compare the outcomes of monotherapy and combination chemotherapy in elderly BTC patients.

## 2. Materials and Methods

### 2.1. Patients

We conducted a retrospective review of consecutive patients with unresectable (locally advanced, metastatic, or recurrent) BTC who received first-line chemotherapy at our institution between 1 January 2016, and 31 December 2021. For the purposes of this study, BTC included intrahepatic and extrahepatic (perihilar or distal) cholangiocarcinomas, gallbladder cancer, and ampullary cancer. While ampullary cancer is sometimes excluded from clinical trials on BTC due to its unique characteristics, it was included in this study as all chemotherapy regimens for BTC received by the study subjects were also indicated for ampullary cancer in Japan. Data were extracted from a prospectively maintained database. Patients enrolled in clinical trials at any time and distal cholangiocarcinoma patients treated with pancreatic cancer regimens due to initial misdiagnosis as pancreatic cancer were excluded from this study.

### 2.2. Baseline Characteristics

Age, ECOG PS, resectability status, presence and location of metastases, and laboratory data, including tumor markers, were evaluated at the time of diagnosis. Patients aged 75 years and older were considered elderly for the purposes of this study, based on the age distribution of BTC and in accordance with the latest proposal from the Japan Gerontological Society and the Japan Geriatrics Society [18] and with recent reports [19,20]. The modified Glasgow prognostic score (mGPS) was calculated based on serum albumin and C-reactive protein (CRP) at diagnosis, scored as 0 if CRP ≤ 1 mg/dL, as 1 if albumin ≥ 3.5 g/dL and CRP ˃ 1 mg/dL, and as 2 if albumin ˂ 3.5 g/dL and CRP ˃ 1 mg/dL [21]. Neutrophil-to-lymphocyte ratio (NLR) was calculated as the ratio of the absolute neutrophil count to the absolute lymphocyte count [22,23].

### 2.3. Chemotherapy

Chemotherapy regimens were selected at the discretion of the oncologist, taking age, general physical condition, cancer status, and other factors into account, and after careful discussions with each patient. Choice of chemotherapy regimen for elderly patients was discussed at department conferences and/or at a multidisciplinary cancer board. Initial dosages were determined based on clinical trials and adjusted for decreased renal function and other relevant factors, but were not reduced solely due to age. Subsequent dosages were reduced based on adverse events, as needed. Adverse events were evaluated based on the National Cancer Institute Common Terminology Criteria for Adverse Events version 4.0 [24]. Chemotherapy was continued until disease progression, patient refusal, intolerable toxicity, conversion surgery, or death.

Contrast-enhanced computed tomography (CT) was performed every 2–3 months, except in cases that developed kidney injury or allergies to contrast media during the follow-up period. Response to chemotherapy was defined as best tumor response on follow-up imaging studies and was evaluated in accordance with the response evaluation criteria in solid tumors (RECIST) guideline (version 1.1) [25]. Overall survival (OS) was defined as the time from the first day of chemotherapy until death from any cause or the last follow-up. Progression-free survival (PFS) was defined as the time from the first day of chemotherapy until death from any cause, disease progression, or the last follow-up. Follow-up data were confirmed up to 31 March 2023.

### 2.4. Statistical Analysis

Categorical variables are shown as absolute numbers and percentages, while continuous variables are shown as medians with ranges. Denominators of ratios were adjusted for missing data. Statistical analyses were conducted using chi-squared or Fisher’s exact tests for categorical variables and the Mann–Whitney U test for continuous variables. Kaplan–Meier and log-rank analyses were conducted to evaluate OS and PFS. Cox regression analysis was conducted to investigate factors associated with OS. Multivariate analysis was performed on variables considered significant in univariate analysis, excluding variables that were not known when chemotherapy was started. *p*-values were two-sided and values < 0.05 were considered statistically significant. All statistical analyses were performed using IBM SPSS Statistics ver. 28.0 (IBM Corp., Armonk, NY, USA).

### 2.5. Ethical Considerations

This study was approved by the Institutional Review Board at our hospital (2023-GB-016). Patient consent was waived due to its retrospective design. The study was publicized on the hospital website, allowing patients to opt out of the study without impacting their care.

## 3. Results

A total of 320 patients commenced first-line chemotherapy at our institution during the study period. We excluded 32 cases that participated in clinical trials and 5 cases initially diagnosed as pancreatic cancer and treated with modified FOLFIRINOX. As a result, 283 patients were included in this study.

### 3.1. Patient Characteristics

Baseline characteristics are shown in Table 1. Elderly patients aged 75 years or older were more likely to have worse ECOG PS compared to the non-elderly group (66.3% vs. 81.7% had ECOG PS of 0), and to have distal cholangiocarcinomas (29.3% vs. 12.6%, *p* < 0.001). Other characteristics were similar between groups.

Elderly patients undergoing monotherapy were older (median of 81 vs. 77 years old, *p* < 0.001) and had worse ECOG PS (55.6% vs. 81.6% had ECOG PS of 0) than those who received combination chemotherapy. The maximum age was 89 years old in the monotherapy group and 82 years old in the combination therapy group. No other significant differences in baseline characteristics were observed.

### 3.2. Treatment-Related Characteristics

Despite less patients receiving combination therapy in the elderly group than in the non-elderly group (41.3% vs. 90.6%, *p* < 0.001), no significant differences in responses to first-line chemotherapy were observed (Table 2). A similar number of patients were able to proceed to second-line chemotherapy (39.1% vs. 40.3%, *p* = 0.849), which involved S-1 monotherapy in over 80% of cases in both groups. There was a tendency for non-elderly patients to undergo conversion surgery (3.3% vs. 8.9%, *p* = 0.083), although the difference was not significant.

Within the elderly group, the monotherapy group tended to have a lower overall response rate (2.2% vs. 14.3%, *p* = 0.081) and to have a lower rate of conversion surgery (0% vs. 7.9%, *p* = 0.067); however, the differences were not significant. The monotherapy group was less likely to proceed to second-line therapy (29.6% vs. 52.6%, *p* = 0.026).

### 3.3. Adverse Events

Adverse events are summarized in Table 3. Elderly patients reported less all-grade constipation and nausea/vomiting but were more likely to experience decreased renal function. Non-elderly patients were more likely to experience severe adverse events (grades 3 or 4; 70.2% vs. 55.4%, *p* = 0.015). Specifically, non-elderly patients experienced more severe episodes of leukopenia, neutropenia, and elevated transaminases.

In the elderly group, patients undergoing monotherapy experienced less all-grade constipation, nausea/vomiting, peripheral neuropathy, and fatigue than those undergoing combination therapy (Table 4). There were no significant differences in severe adverse events between groups (50.0% vs. 63.2%, *p* = 0.211).

### 3.4. Factors Affecting Survival

**Figure 1:**
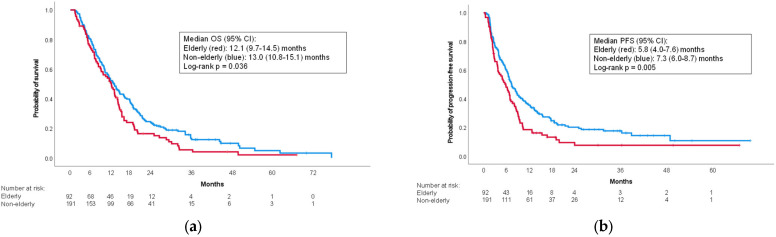
Kaplan–Meier curves for the overall cohort. (**a**) Overall survival; (**b**) progression-free survival. CI: confidence interval; OS: overall survival; PFS: progression-free survival.

The elderly group had a slightly shorter median OS than the non-elderly group (12.2 (95% confidence interval (CI): 9.7–14.5) months vs. 13.0 (95% CI: 10.8–15.1) months; *p* = 0.036) (Figure 1a). Median PFS was also shorter in the elderly group (5.8 (95% CI: 4.0–7.6) months vs. 7.3 (95% CI: 6.0–8.7) months; *p* = 0.005) (Figure 1b).

**Figure 2:**
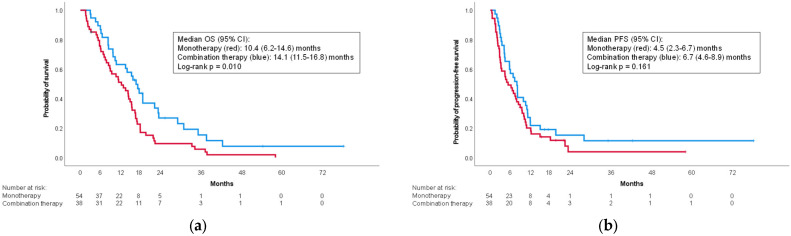
Kaplan–Meier curves for the elderly cohort. (**a**) Overall survival; (**b**) progression-free survival. CI: confidence interval; OS: overall survival; PFS: progression-free survival.

Within the elderly group, median OS in the monotherapy group was shorter than the combination therapy group (10.4 (95% CI: 6.2–14.6) months vs. 14.1 (95% CI: 11.5–16.8) months; *p* = 0.010) (Figure 2a). The difference in median PFS was not significant (4.5 (95% CI: 2.3–6.7) months vs. 6.7 (95% CI: 4.6–8.9) months; *p* = 0.161) (Figure 2b).

An age of 75 years or older was a significant predictor of OS in the overall cohort in the univariate analysis (hazard ratio (HR): 1.33; *p* = 0.039) but did not remain significant in the multivariate analysis (Table 5). Multivariate Cox regression analyses revealed that NLR values less than 3, mGPS of 0, normal carcinoembryonic antigen (CEA), and choice of triplet therapy with gemcitabine, cisplatin, and S-1 were significant predictors of longer OS.

In the elderly cohort, choice of monotherapy was significantly associated with shorter OS (HR: 1.78, *p* = 0.012), but did not remain significant in multivariate analysis (Table 6). Only mGPS values of 1 or 2 and a CEA of 5 of more were significant predictors of shorter OS.

An age of 75 years or older was a significant predictor of shorter PFS in the overall cohort, in both univariate (HR: 1.47; *p* = 0.006) and multivariate analyses (HR: 1.44; *p* = 0.029) (Table 7). Other significant independent predictors of shorter PFSs were existence of liver metastases, existence of lung metastases, and NLR of 3 or more.

In the elderly cohort, monotherapy was not a significant predictor of PFS (Table 8). Significant independent predictors of shorter PFS were existence of liver metastases, existence of lung metastases, mGPS values of 1 or 2, and elevated CEA.

## 4. Discussion

In this study, we conducted a retrospective review of chemotherapy for BTC patients in the pre-immune checkpoint inhibitor (ICI) era, with a focus on safety and efficacy of palliative chemotherapy in elderly patients. We found that elderly patients had worse ECOG PS and were more likely to receive monotherapy than the non-elderly group; however, the response rates were similar. Both OS and PFS were longer in the non-elderly group; however, severe adverse events were also more frequent. Age was an independent predictor of PFS but not of OS. Within the elderly cohort, patients receiving monotherapy were less likely to proceed to second-line treatment than those receiving combination therapy. There were no differences in severe adverse events between groups. Median OS, but not PFS, was longer in the combination therapy group; however, choice of monotherapy was not an independent predictor of either OS or PFS in the multivariate analyses.

Despite the lack of an upper age limit in most recent prospective studies, elderly patients are grossly underrepresented (Table 9). While a poor PS is more common in the elderly, it is difficult to deny that trial investigators are reluctant to enter even healthy octogenarians into clinical trials. Patients aged 65 years or older, 70 years or older, and 75 years or older made up 74%, 54%, and 33% of the patients in our real-world study, respectively, while 32–64% were aged 65 years or older and 0–17% were aged 75 years or older in prospective studies. We conducted chemotherapy in BTC patients as old as 89 years, while the maximum age from the ten major evaluated studies was 84 years.

Gemcitabine monotherapy has been reported to be similarly safe and effective in elderly BTC patients, with cutoffs set at 70 [26] and 75 [20] years of age. With respect to combination therapy, patients aged 70 years or older predicted poor prognoses in a study on BTC patients receiving gemcitabine and S-1 (GS) [27]. On the other hand, age was not a significant predictor of survival in BTC patients receiving gemcitabine and cisplatin (GC) [28]. An analysis of patients receiving either GC or GS in a clinical trial revealed no significant differences in survival or adverse events based on age with a cutoff of 75 years old, although the elderly group only included patients aged 75–79 years old [19].

A long review of studies comparing elderly and non-elderly BTC patients undergoing chemotherapy, including a subgroup analysis of the ABC-02 trial [8], found that age had no impact on either OS or PFS, regardless of whether monotherapy or combination therapy was provided [29]. The same study found that combination therapy achieved higher OS (HR: 0.54, *p* = 0.001) and PFS (HR: 0.60, *p* = 0.004) results in a subgroup of patients aged 70 years old or older.

We found that, while OS and PFS were longer in non-elderly patients, they also experienced more severe adverse events. As severe adverse events can lead to rapid decline in ECOG PS and often lead to the termination of chemotherapy in the elderly, physician judgment in choosing between monotherapy and combination therapy is crucial to maximize OS while maintaining quality of life. Age was not an independent predictor of OS, consistent with previous prospective studies [30], even when physician discretion was introduced. Our study indicated that metrics, such as NLR, mGPS, and CEA, were better predictors of OS than age, implying that judicious selection of chemotherapy regimens can contribute to the achievement of OS in elderly patients comparable to that of non-elderly patients.

Our investigation of differences in outcomes between monotherapy and combination therapy in elderly BTC patients also shed light on the optimal treatment strategy in this population. Specifically, choice of monotherapy was not an independent predictor of neither OS nor PFS in multivariate analysis. Starting with monotherapy also allowed almost 40% of patients to proceed to second-line chemotherapy, which is similar to the percentage of non-elderly patients who received second-line chemotherapy. On the other hand, three elderly patients received combination therapy and went on to receive conversion surgery, achieving prolonged OS. Thus, combination therapy may not necessarily be preferable to monotherapy in patients aged 75 years and over; however, some fit patients, such as those who were candidates for conversion therapy, may have benefitted from aggressive combination therapy. Elderly patients with good PS and favorable baseline characteristics predicting longer PFS in this study (absence of liver or lung metastases, NLR < 3, and CEA < 5) may also benefit from combination therapy, regardless of age. A trial comparing combination therapy at a reduced dose to full-dose monotherapy is ongoing for elderly patients with pancreatic cancer [31], and similar studies may be beneficial for elderly BTC patients.

Systemic therapy for BTC is undergoing a paradigm shift towards ICIs and targeted therapy at present, with many clinical trials underway [32]. Data on elderly patients gained in this study may serve as a comparison arm for future real-world analyses involving such new agents. While treatment for BTC has finally entered the ICI era with the TOPAZ-1 trial [15], various questions remain unanswered. For example, it remains unclear whether elderly patients should be given GC at a reduced dose to allow for combination therapy with ICIs [15,17]. Another alternative to be considered is whether or not gemcitabine monotherapy can be combined with ICIs in elderly patients. As most prospective studies have neglected to perform subgroup analyses based on age [26], more research is needed to serve unmet needs for safe and effective treatment in elderly BTC patients.

This study had several limitations. This was a single-center, retrospective study with inherent selection bias. BTC is a heterogeneous disease with varying characteristics, limiting the applicability of our results to underrepresented cancer types, such as ampullary cancer. Analyses of co-morbidities and geriatric assessment were not conducted. Data on relative dose intensities were not available. The inclusion of patients receiving S-1 may limit the generalizability of our results to non-Asian countries where the drug is not the standard of care or is not available.

## 5. Conclusions

Despite the use of different chemotherapy regimens, age was not an independent predictor of OS in patients undergoing chemotherapy for BTC. Use of monotherapy vs. combination therapy also did not independently predict OS in BTC patients aged 75 years old or older. While monotherapy appears to be a viable alternative in elderly BTC patients, treatment should be tailored to the individual. Ongoing and future studies involving ICIs and targeted agents may provide safer and more tolerable options for this population.

## Figures and Tables

**Table 1:** Baseline characteristics.

						Elderly Patients				
	Non-Elderly		Elderly			Monotherapy		Combination		
	(*n* = 191)		(*n* = 92)		*p*-Value	(*n* = 54)		(*n* = 38)		*p*-Value
Age in years, median (range)	67	(24–74)	78.5	(75–89)	<0.001	81	(75–89)	77	(75–82)	<0.001
Male (*n*, %)	116	60.7%	55	59.8%	0.878	35	64.8%	20	52.6%	0.241
Body mass index, median (range)	20.8	(13.6–34.4)	20.6	(14.1–29.5)	0.352	20.5	(14.9–28.9)	21.7	(14.1–29.5)	0.168
Performance status, 0/1/2	156/34/1		61/29/2		0.013	30/22/2		31/7/0		0.027
Primary cancer (*n*, %)										
Intrahepatic	48	25.1%	15	16.3%	0.095	9	16.7%	6	15.8%	0.911
Extrahepatic (perihilar)	51	26.7%	28	30.4%	0.512	18	33.3%	10	26.3%	0.471
Extrahepatic (distal)	24	12.6%	27	29.3%	<0.001	13	24.1%	14	36.8%	0.185
Gallbladder	51	26.7%	18	19.6%	0.190	13	24.1%	5	13.2%	0.194
Ampulla	17	8.9%	4	4.3%	0.171	1	1.9%	3	7.9%	0.303
Cancer status (*n*, %)										
Locally advanced	33	17.3%	13	14.1%	0.501	5	9.3%	8	21.1%	0.110
Metastatic	96	50.3%	42	45.7%	0.467	26	48.1%	16	42.1%	0.567
Recurrent	62	32.5%	37	40.2%	0.200	23	42.6%	14	36.8%	0.580
Location of metastases ^1^										
Liver	68	35.6%	32	34.8%	0.893	21	38.9%	11	28.9%	0.324
Lung	25	13.1%	13	14.1%	0.810	5	9.3%	8	21.1%	0.110
Lymph nodes	62	32.5%	25	27.2%	0.367	15	27.8%	10	26.3%	0.877
Peritoneal dissemination	52	27.2%	23	25.0%	0.691	14	25.9%	9	23.7%	0.807
Bone	6	3.1%	1	1.1%	0.297	1	1.9%	0	0.0%	>0.999
Laboratory data										
Modified Glasgow prognostic score, 0/1/2	127/32/32		52/17/23		0.207	27/11/16		25/6/7		0.308
Neutrophil-to-lymphocyte ratio, median (range)	2.5	(0.4–30.7)	2.7	(0.7–14.0)	0.955	2.8	(0.7–14.0)	2.6	(1.6–12.3)	0.943
CEA, ng/mL, median (range)	3.3	(0.5–1398)	4.0	(1.1–386)	0.238	4.0	(1.1–386)	4.0	(1.2–358)	0.643
CA19-9, U/mL, median (range)	159	(2–50,000)	177	(2–50,000)	0.739	159	(2–50,000)	177	(2–50,000)	0.883

CA19-9: carbohydrate antigen 19-9; CEA: carcinoembryonic antigen. ^1^ Some patients had metastases to multiple locations, while others had none.

**Table 2:** Treatment-related characteristics.

						Elderly Patients				
(*n*, %)	Non-Elderly		Elderly			Monotherapy		Combination		
	(*n* = 191)		(*n* = 92)		*p*-Value	(*n* = 54)		(*n* = 38)		*p*-Value
First-line chemotherapy	191	100.0%	92	100.0%	-	54	100.0%	38	100.0%	
Combination therapy	173	90.6%	38	41.3%	<0.001					
Gemcitabine + cisplatin + S-1	19	9.9%	1	1.1%	0.006			1	2.6%	
Gemcitabine + cisplatin	150	78.5%	34	37.0%	<0.001			34	89.5%	
Gemcitabine + S-1	4	2.1%	3	3.3%	0.686			3	7.9%	
Monotherapy	18	9.4%	54	58.7%	<0.001					
Gemcitabine	17	8.9%	36	39.1%	<0.001	36	66.7%			
S-1	1	0.5%	18	19.6%	<0.001	18	33.3%			
Response to first-line chemotherapy										
Complete response	1	0.5%	0	0.0%	>0.999	0	0.0%	0	0.0%	-
Partial response	22	11.5%	6	6.5%	0.187	1	1.9%	5	13.2%	0.078
Stable disease	112	58.6%	48	52.2%	0.304	26	48.1%	22	57.9%	0.357
Progressive disease	40	20.9%	26	28.3%	0.173	18	33.3%	8	21.1%	0.198
Not evaluated	16	8.4%	12	13.0%	0.218	9	16.7%	3	7.9%	0.347
Overall response rate		13.1%		7.5%	0.188		2.2%		14.3%	0.081
Disease control rate		77.1%		67.5%	0.103		60.0%		77.1%	0.104
Reason for termination of first-line chemotherapy										
Disease progression	144	75.4%	77	83.7%	0.114	45	83.3%	32	84.2%	0.911
Intolerance	11	5.8%	6	6.5%	0.800	5	9.3%	1	2.6%	0.395
Conversion surgery	17	8.9%	3	3.3%	0.083	0	0.0%	3	7.9%	0.067
Patient refusal	7	3.7%	1	1.1%	0.220	1	1.9%	0	0.0%	>0.999
Treatment ongoing	2	1.0%	1	1.1%	>0.999	0	0.0%	1	2.6%	0.413
Other	10	5.2%	4	4.3%		3	5.6%	1	2.6%	
Second-line chemotherapy	77	40.3%	36	39.1%	0.849	16	29.6%	20	52.6%	0.026
Gemcitabine + cisplatin	5	2.6%	2	2.2%		0	0.0%	2	5.3%	0.168
Gemcitabine + S-1	4	2.1%	1	1.1%		0	0.0%	1	2.6%	0.413
Gemcitabine	0	0.0%	2	2.2%		2	3.7%	0	0.0%	0.510
S-1	64	33.5%	31	33.7%		14	25.9%	17	44.7%	0.060
Other	4	2.1%	0	0.0%		0	0.0%	0	0.0%	-

**Table 3:** Adverse events—overall cohort.

	All Grades					Grades 3–4				
(*n*, %)	Non-Elderly		Elderly			Non-Elderly		Elderly		
	(*n* = 191)		(*n* = 92)		*p*-Value	(*n* = 191)		(*n* = 92)		*p*-Value
All adverse events	191	100.0%	92	100.0%	-	134	70.2%	51	55.4%	0.015
Hematologic adverse events										
Leukopenia	141	73.8%	60	65.2%	0.135	55	28.8%	12	13.0%	0.004
Neutropenia	152	79.6%	67	72.8%	0.203	103	53.9%	33	35.9%	0.004
Anemia	186	97.4%	90	97.8%	>0.999	42	22.0%	19	20.7%	0.798
Thrombocytopenia	148	77.5%	70	76.1%	0.793	14	7.3%	6	6.5%	0.804
Febrile neutropenia	0	0.0%	0	0.0%	-	0	0.0%	0	0.0%	-
Non-hematologic adverse events										
Stomatitis	45	23.6%	9	9.8%	0.006	1	0.5%	0	0.0%	>0.999
Decreased appetite	28	14.7%	21	22.8%	0.089	1	0.5%	1	1.1%	0.545
Diarrhea	32	16.8%	16	17.4%	0.894	0	0.0%	0	0.0%	-
Constipation	153	80.1%	54	58.7%	<0.001	0	0.0%	0	0.0%	-
Nausea/vomiting	107	56.0%	30	32.6%	<0.001	1	0.5%	0	0.0%	>0.999
Peripheral neuropathy	64	33.5%	14	15.2%	0.001	0	0.0%	0	0.0%	-
Alopecia	9	4.7%	5	5.4%	0.776	0	0.0%	0	0.0%	-
Fatigue	166	86.9%	70	76.1%	0.022	1	0.5%	2	2.2%	0.248
Elevated transaminases	173	90.6%	82	89.1%	0.703	25	13.1%	5	5.4%	0.050
Decreased renal function	35	18.3%	28	30.4%	0.022	0	0.0%	1	1.1%	0.325
Interstitial pneumonitis	0	0.0%	2	2.2%	0.105	0	0.0%	2	2.2%	0.105
Rash	43	22.5%	17	18.5%	0.437	1	0.5%	0	0.0%	>0.999

**Table 4:** Adverse events—elderly cohort.

	All Grades					Grades 3–4				
(*n*, %)	Mono- Therapy		Combination			Mono- Therapy		Combination		
	(*n* = 54)		(*n* = 38)		*p*-Value	(*n* = 54)		(*n* = 38)		*p*-Value
All adverse events	54	100.0%	38	100.0%	-	27	50.0%	24	63.2%	0.211
Hematologic adverse events										
Leukopenia	31	57.4%	29	76.3%	0.061	6	11.1%	6	15.8%	0.543
Neutropenia	36	66.7%	31	81.6%	0.113	16	29.6%	17	44.7%	0.137
Anemia	52	96.3%	38	100.0%	0.510	9	16.7%	10	26.3%	0.260
Thrombocytopenia	40	74.1%	30	78.9%	0.589	2	3.7%	4	10.5%	0.226
Febrile neutropenia	0	0.0%	0	0.0%	-	0	0.0%	0	0.0%	-
Non-hematologic adverse events										
Stomatitis	5	9.3%	4	10.5%	>0.999	0	0.0%	0	0.0%	-
Decreased appetite	12	22.2%	9	23.7%	0.869	0	0.0%	1	2.6%	0.413
Diarrhea	6	11.1%	10	26.3%	0.058	0	0.0%	0	0.0%	-
Constipation	23	42.6%	31	81.6%	<0.001	0	0.0%	0	0.0%	-
Nausea/vomiting	12	22.2%	18	47.4%	0.011	0	0.0%	0	0.0%	-
Peripheral neuropathy	4	7.4%	10	26.3%	0.013	0	0.0%	0	0.0%	-
Alopecia	1	1.9%	4	10.5%	0.156	0	0.0%	0	0.0%	-
Fatigue	34	63.0%	36	94.7%	<0.001	0	0.0%	2	5.3%	0.168
Elevated transaminases	47	87.0%	35	92.1%	0.515	4	7.4%	1	2.6%	0.400
Decreased renal function	19	35.2%	9	23.7%	0.238	1	1.9%	0	0.0%	>0.999
Interstitial pneumonitis	1	1.9%	1	2.6%	>0.999	1	1.9%	1	2.6%	>0.999
Rash	9	16.7%	8	21.1%	0.594	0	0.0%	0	0.0%	-

**Table 5:** Factors affecting overall survival—overall cohort.

	Univariate			Multivariate (Predictors Only)		
	Hazard Ratio	95% CI	*p*-Value	Hazard Ratio	95% CI	*p*-Value
Baseline characteristics						
Male sex	0.98	0.76–1.27	0.904			
Elderly (75 years or older)	1.33	1.02–1.73	0.039	1.07	0.78–1.48	0.669
Performance status (1 or 2)	1.51	1.12–2.02	0.008	1.03	0.74–1.44	0.859
Tumor characteristics						
Locally advanced (vs. metastatic or recurrence)	0.58	0.40–0.84	0.003	0.93	0.59–1.49	0.769
Gallbladder cancer	1.57	1.18–2.09	0.002	1.30	0.94–1.79	0.114
Extrahepatic (perihilar) cholangiocarcinoma	0.80	0.60–1.06	0.120	1.04	0.75–1.45	0.808
Liver metastasis	1.33	1.03–1.73	0.031	1.40	1.02–1.92	0.038
Lung metastasis	1.34	0.94–1.91	0.108			
Lymph node metastasis	1.37	1.05–1.79	0.021	1.28	0.94–1.74	0.113
Peritoneal dissemination metastasis	1.48	1.12–1.96	0.006	1.36	0.99–1.86	0.057
Bone metastasis	2.11	0.99–4.50	0.052			
Laboratory values						
Neutrophil-to-lymphocyte ratio (3 or more)	1.69	1.31–2.17	<0.001	1.57	1.20–2.04	<0.001
mGPS (1 or 2)	1.87	1.45–2.42	<0.001	1.65	1.25–2.17	<0.001
CEA (5 ng/mL or more)	2.06	1.59–2.65	<0.001	1.73	1.32–2.27	<0.001
CA19-9 (500 U/mL or more)	1.48	1.15–1.92	0.003	1.18	0.90–1.55	0.243
Treatment						
Monotherapy	1.73	1.31–2.28	<0.001	1.39	0.97–1.99	0.074
First-line GCS	0.39	0.20–0.76	0.005	0.43	0.22–0.85	0.016
Any second-line chemotherapy	0.69	0.54–0.89.	0.005			
Conversion surgery	0.21	0.11–0.40	<0.001			

CA19-9: carbohydrate antigen; CEA: carcinoembryonic antigen; CI: confidence interval; GCS: gemcitabine + cisplatin + S-1 triplet chemotherapy; mGPS: modified Glasgow prognostic score.

**Table 6:** Factors affecting overall survival—elderly cohort.

	Univariate			Multivariate (Predictors only)		
	Hazard Ratio	95% CI	*p*-Value	Hazard Ratio	95% CI	*p*-Value
Baseline characteristics						
Male sex	1.01	0.65–1.57	0.980			
Performance status (1 or 2)	1.13	0.72–1.78	0.587			
Tumor characteristics						
Recurrence	1.21	0.78–1.88	0.386			
Extrahepatic (perihilar) cholangiocarcinoma	0.57	0.35–0.94	0.021	1.01	0.62–1.66	0.971
Liver metastasis	1.80	1.14–2.84	0.140			
Lung metastasis	1.42	0.76–2.63	0.268			
Lymph node metastasis	1.19	0.74–1.92	0.470			
Peritoneal dissemination metastasis	1.12	0.68–1.82	0.667			
Bone metastasis	3.41	0.46–25.2	0.230			
Laboratory values						
Neutrophil-to-lymphocyte ratio (3 or more)	1.59	1.03–2.45	0.036	1.28	0.80–2.04	0.303
mGPS (1 or 2)	2.35	1.51–3.67	<0.001	2.24	1.42–3.54	<0.001
CEA (5 ng/mL or more)	1.87	1.20–2.92	0.006	1.70	1.03–2.79	0.036
CA19-9 (37 U/mL or more)	0.90	0.56–1.43	0.651			
Treatment						
Monotherapy	1.78	1.14–2.78	0.012	1.48	0.93–2.35	0.102
Any second-line chemotherapy	0.62	0.40–0.97	0.036			
Conversion surgery	0.12	0.17–0.90	0.040			

CA19-9: carbohydrate antigen; CEA: carcinoembryonic antigen; CI: confidence interval; GCS: gemcitabine + cisplatin + S-1 triplet chemotherapy; mGPS: modified Glasgow prognostic score.

**Table 7:** Factors affecting progression-free survival—overall cohort.

	Univariate			Multivariate		
	Hazard Ratio	95% CI	*p*-Value	Hazard Ratio	95% CI	*p*-Value
Baseline characteristics						
Male sex	1.01	0.78–1.32	0.916			
Elderly (75 years or older)	1.47	1.12–1.94	0.006	1.44	1.04–1.99	0.029
Performance status (1 or 2)	1.21	0.89–1.65	0.233			
Tumor characteristics						
Locally advanced (vs. metastatic or recurrence)	0.47	0.31–0.70	<0.001	0.69	0.42–1.18	0.130
Gallbladder cancer	1.45	1.07–1.92	0.017	1.21	0.87–1.68	0.252
Liver metastasis	1.49	1.13–1.96	0.005	1.47	1.06–2.04	0.023
Lung metastasis	1.60	1.11–2.29	0.016	1.69	1.15–2.47	0.007
Lymph node metastasis	1.33	1.01–1.76	0.041	1.25	0.91–1.70	0.167
Peritoneal dissemination metastasis	1.36	1.03–1.82	0.034	1.28	0.93–1.77	0.133
Bone metastasis	1.51	0.67–3.39	0.325			
Laboratory values						
Neutrophil-to-lymphocyte ratio (3 or more)	1.69	1.30–2.20	<0.001	1.64	1.24–2.16	<0.001
mGPS (1 or 2)	1.63	1.25–2.14	<0.001	1.31	0.98–1.75	0.072
CEA (5 ng/mL or more)	1.59	1.22–2.07	<0.001	1.27	0.96–1.69	0.101
CA19-9 (500 ng/mL or more)	1.55	1.18–2.02	<0.001	1.29	0.97–1.72	0.084
Treatment						
Monotherapy	1.59	1.19–2.13	0.002	1.20	0.85–1.69	0.313
First-line GCS	0.52	0.29–0.93	0.028	0.56	0.30–1.02	0.060

CA19-9: carbohydrate antigen; CEA: carcinoembryonic antigen; CI: confidence interval; GCS: gemcitabine + cisplatin + S-1 triplet chemotherapy; mGPS: modified Glasgow prognostic score.

**Table 8:** Factors affecting progression-free survival—elderly cohort.

	Univariate			Multivariate (Predictors Only)		
	Hazard Ratio	95% CI	*p*-Value	Hazard Ratio	95% CI	*p*-Value
Baseline characteristics						
Male sex	1.17	0.75–1.83	0.494			
Performance status (1 or 2)	0.97	0.61–1.57	0.914			
Tumor characteristics						
Locally advanced (vs. metastatic or recurrence)	0.40	0.20–0.80	0.010	0.64	0.28–1.43	0.274
Extrahepatic (perihilar) cholangiocarcinoma	0.46	0.43–0392	0.022	0.67	0.39–1.15	0.150
Liver metastasis	2.61	1.63–4.19	<0.001	2.10	1.26–3.47	0.003
Lung metastasis	2.40	1.27–4.52	0.007	3.10	1.54–6.19	0.001
Lymph node metastasis	1.01	0.62–1.65	0.977			
Peritoneal dissemination metastasis	1.14	0.70–1.87	0.595			
Bone metastasis	2.81	0.38–20.7	0.310			
Laboratory values						
Neutrophil-to-lymphocyte ratio (3 or more)	1.58	1.02–2.46	0.042	1.10	0.66–1.85	0.715
mGPS (1 or 2)	1.69	1.07–2.66	0.023	2.07	1.24–3.45	0.006
CEA (5 ng/mL or more)	2.52	1.59–4.01	<0.001	1.87	1.05–3.35	0.035
CA19-9 (37 ng/mL or more)	0.97	0.60–1.57	0.901			
Treatment						
Monotherapy	1.38	0.88–2.16	0.163			

CA19-9: carbohydrate antigen; CEA: carcinoembryonic antigen; CI: confidence interval; GCS: gemcitabine + cisplatin + S-1 triplet chemotherapy; mGPS: modified Glasgow prognostic score.

**Table 9:** Elderly patient participation in major recent prospective studies.

									% Aged:			
Trial Name	Year	Phase	Treatment	Line	*n*	Upper Age Limit (Inclusion Criteria)	Oldest (Years)	Median Age	≥65	≥70	≥75	PS
FUGA-BT [7]	2019	III	GC vs. GS	1	354	79	79	67/67	64%		17%	0–1
PRODIGE 12-ACCORD 18-UNICANCER GI [9]	2019	III	Gemcitabine/Oxaliplatin vs. observation	Adjuvant	196	None	83	63/63				0–2
BILCAP [10]	2019	III	Capecitabine vs. observation	Adjuvant	447	None	69	62/64		0%	0%	0–1
ClarIDHy [11]	2020	III	Ivodenib vs. placebo	2 or 3	185	None	83	61/63				0–1
FIGHT-202 [12]	2020	III	Pemigatinib	2	146	None	78	59	32%		8%	0–2
ABC-06 [13]	2021	III	FOLFOX vs. ASC	2	162	None	84	65/65	50%			0–2
NIFTY [14]	2021	IIb	5-FU/LV ± Nanoliposomal irinotacan	2	174	None	84	63/65	48%			0–1
TOPAZ-1 [15]	2022	III	GC ± Durvalumab	1	685	None	85	64/64	47%			0–1
KHBO1401-MITSUBA [16]	2023	III	GC ± S-1	1	246	None	84	68/68				0–2
KEYNOTE-966 [17]	2023	III	GC ± Pembrolizumab	1	1069	None	71	64/63	47%			0–1
(This study)	2023	-	GCS, GC, GS, Gemcitabine, S-1	1	283	None	89	70	74%	54%	33%	0–2

ASC: active symptom control; GC: gemcitabine + cisplatin; GCS: gemcitabine + cisplatin + S-1 triplet chemotherapy; GS: gemcitabine + S-1, PS: performance status.

## Data Availability

Data are available from the corresponding author upon reasonable request.
